# Relationship between maternal lipid profile during the third trimester and the risk of small-for-gestational-age birth: exploring inadequate gestational weight gain as a mediator

**DOI:** 10.1186/s12944-026-02865-x

**Published:** 2026-01-12

**Authors:** Maissam Ghanem, Weiming Wang, Huaqi Zhang, Jin Liu, Qian Liang, Nianhong Yang

**Affiliations:** Hubei Key Laboratory of Food Nutrition and Safety, MOE Key Laboratory of Environment and Health, Department of Nutrition and Food Hygiene, School of Public Health, Tongji Medical College, Huazhong University of Science & Technology, Wuhan, 430030 China

**Keywords:** Small-for-gestational-age, Triglycerides, High-density lipoprotein cholesterol, Gestational weight gain, Pregnancy

## Abstract

**Background:**

Lipids play crucial roles in maternal and foetal metabolism; however, their effects on birth weight remain unclear. Moreover, the potential mediating role of inadequate gestational weight gain (iGWG) in this association remains unexplored. The objective of this study was to assess maternal lipid profiles in the third trimester and their associations with the risk of small-for-gestational-age (SGA) infants, focusing on the combined effects of high-density lipoprotein cholesterol (HDL-C) and triglycerides (TG). This study also examined whether iGWG influenced this relationship.

**Methods:**

Data were sourced from the Tongji Maternal and Child Health Cohort. Maternal fasting lipid levels were measured during the third trimester and birth information was retrieved from medical records. Log-Poisson regression analysis was performed to assess the relationship between lipid tertiles and SGA risk. The possible mediating role of iGWG was examined using the *mediation* package in R.

**Results:**

An increased risk of SGA was associated with high HDL-C levels (adjusted relative risk [aRR], 1.54; 95% confidence interval [CI], 1.15–2.07), particularly among mothers with high HDL-C and low TG levels (aRR, 1.77; 95% CI, 1.10–2.87). This association remained significant among individuals with normal pre-pregnancy weight. The relationship between lipid profiles and SGA was independent of iGWG.

**Conclusions:**

An increased risk of SGA was associated with high maternal HDL-C levels. The combination of low TG and high HDL-C levels was identified as a significant predictor of SGA. iGWG did not explain these associations.

**Supplementary Information:**

The online version contains supplementary material available at 10.1186/s12944-026-02865-x.

## Background

Pregnancy induces profound changes in the body, primarily driven by the growing demand for metabolic fuels required to support foetal growth and development. Concurrently, hormonal changes lead to modifications in the lipid profile across pregnancy trimesters [1]. Between weeks 9 and 13, lipid components, including total cholesterol (TC), triglycerides (TG), high-density lipoprotein cholesterol (HDL-C), and low-density lipoprotein cholesterol (LDL-C), begin to rise and continue to increase gradually as pregnancy progresses [2].

Disruptions in maternal lipid metabolism, including those associated with gestational diabetes mellitus [3] and preeclampsia, have been reported as potential contributors to unfavourable pregnancy outcomes [4]. These disturbances may jeopardise foetal growth, thereby increasing the risk of adverse birth outcomes [5, 6]. Among these, small-for-gestational-age (SGA) at birth poses a critical challenge to infant health and has been linked to an increased risk of neonatal mortality [7]. SGA reflects impaired foetal growth and is associated with perinatal complications such as hyperbilirubinemia and hypoglycaemia. Additionally, it may lead to long-term consequences, including poor academic performance in adolescence and an increased risk of metabolic syndrome in adulthood [8, 9]. Evidence indicates that children born SGA, even those who subsequently achieved normal weight in early childhood, commonly exhibit abnormal lipid profiles [10], which may predispose them to future cardiovascular disorders.

The associations of SGA with smoking [11], low pre-pregnancy body mass index (BMI) [12], and reduced height thresholds have been well characterised [13]. Moreover, gestational dyslipidaemia has been identified as a potential risk factor for SGA [14]. The substantial risk of SGA, along with its largely irreversible nature, underscores the urgent need for effective strategies to predict and prevent its occurrence.

Birth weight and gestational weight gain (GWG) are closely linked, as GWG is a key indicator of maternal nutritional status throughout pregnancy. Adequate GWG is crucial for foetal growth and development, whereas inadequate GWG (iGWG) has been associated with unfavourable delivery outcomes, including low birth weight, SGA, and preterm birth [15]. Additionally, associations between GWG and altered lipid profiles, placental weight, and placenta-to-birth weight have been documented [16]. However, whether iGWG mediates the relationship between altered lipid profiles and birth outcomes remains unclear. It is hypothesised that iGWG mediates the association between lipid profile disturbances and SGA birth. Understanding this relationship is vital for improving prenatal care strategies and supporting risk mitigation.

The present study aimed to (1) explore the association between maternal lipid profiles during the third trimester and SGA risk, with a focus on the combined effects of HDL-C and TG levels, and (2) investigate whether iGWG mediates this association.

## Methods

### Study population and design

Data were obtained from the Tongji Maternal and Child Health Cohort (TMCHC), a prospective cohort study designed to investigate the effects of maternal lifestyle and nutrition on maternal and neonatal outcomes in Wuhan, Hubei Province, China [17]. Recruitment was conducted at three research hospitals in Wuhan from January 2013 to May 2016. Pregnant women attending their first antenatal visit before 16 weeks of gestation were enrolled and followed up at regular intervals throughout pregnancy.

Participants were eligible if they met the following criteria: (1) singleton pregnancy; (2) provision of blood samples during the third trimester for lipid assessment; and (3) availability of follow-up data on birth outcomes. Women with multiple gestations and pre-existing conditions, such as diabetes, hypertension, thyroid disorders, and known infectious diseases (e.g. viral hepatitis, tuberculosis, etc.) were excluded. A flowchart of participant selection is provided in Supplementary Fig. S1.

### Lipid assessment

Throughout the third trimester, fasting maternal blood samples were collected by trained nurses. TG, TC, LDL-C, and HDL-C were measured using the glycerol phosphate oxidase-p-aminophenazone (GPO-PAP), cholesterol oxidase–peroxidase and 4-aminoantipyrine phenol (CHOD-PAP), surfactant scavenging, and catalase scavenging methods, respectively. All measurements were performed using a Mindray BS-200 automatic biochemical analyser (Shenzhen, China) in accordance with standard procedures.

### Birth outcomes

Data on newborn characteristics, including birth weight, sex, and gestational age at delivery (in weeks), were obtained from medical records. Infants with birth weights below the 10th percentile for sex- and gestational-age–specific standards were classified as SGA, according to Chinese reference standards [18].

### Covariates

At enrolment, data on maternal obstetric and medical histories, socioeconomic status, and lifestyle characteristics were collected using a face-to-face questionnaire. Recorded variables included maternal age, educational level, parity, ethnicity, smoking habits, average monthly income, physical activity, and alcohol consumption. Moderate-intensity physical activity (≥ 30 min, at least three times per week) was considered regular during pregnancy.

Gestational age was initially estimated using the date of the last menstrual period and was adjusted using ultrasonography when discrepancies were identified during early pregnancy. Pre-pregnancy BMI was calculated as the weight before pregnancy (kg) divided by the square of the height (m^2^). According to the Working Group on Obesity in China, participants were classified into four BMI categories: (1) underweight, BMI < 18.5 kg/m^2^; (2) normal weight, 18.5 ≤ BMI < 24.0 kg/m^2^; (3) overweight, 24.0 ≤ BMI < 28.0 kg/m^2^; and (4) obese, BMI ≥ 28.0 kg/m^2^ [19]. Pre-pregnancy weight was self-reported, whereas height and weight were measured at enrolment.

Total GWG was calculated by subtracting the pre-pregnancy weight from the weight recorded before delivery. GWG was classified according to the recommended ranges for Chinese women across BMI categories [20]: 11.0–16.0 kg for individuals classified as underweight; 8.0–14.0 kg for those within the normal weight range; 7.0–11.0 kg for those classified as overweight; and 5.0–9.0 kg for those classified as obese. GWG was further categorised as inadequate (below the recommended range), adequate (within the range), and excessive (above the range).

### Statistical analysis

Continuous variables with normal distributions are summarised as mean ± standard deviation (SD), whereas non-normally distributed variables are presented as median (interquartile range [IQR]). Categorical variables are presented as frequencies (*n*) and percentages (%). Log-Poisson regression analysis was performed to examine the association between lipid levels and risk of SGA. Relative risks (RRs) and adjusted RRs (aRRs), along with 95% confidence intervals (CIs), were calculated. The RR of SGA was estimated across lipid-level tertiles, with the middle tertile (T2) as the reference group.

**Fig. 1:**
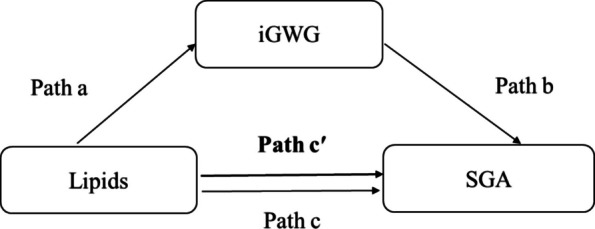
Mediation diagram illustrating the role of iGWG in the association between maternal lipid levels and SGA. Path a represents the association between lipid levels and iGWG (log-Poisson regression). Path b represents the association between iGWG and SGA (log-Poisson regression). Path c represents the association between lipid levels and SGA without adjusting for iGWG (log-Poisson regression). Path c': represents the association between lipid levels and SGA after adjusting for iGWG (log-Poisson regression). iGWG: inadequate gestational weight gain, SGA: small-for-gestational-age

Models were adjusted for the following covariates: maternal ethnicity (Han Chinese or other), age, parity (primiparous or multiparous), pre-pregnancy BMI, regular physical activity in early pregnancy (yes/no), educational level (< 12 years, 12–15, ≥ 16 years according to the number of completed schooling), monthly average income (< 5000, 5000–9999 or ≥ 10,000 Chinese Yuan), gestational age at blood sampling, neonatal sex (male/female), and total weight gain. Data analyses were performed using IBM SPSS Statistics version 26.0 for Windows (IBM Corp., Armonk, NY, USA). Mediation analysis was conducted following the steps described by Baron and Kenny to evaluate whether iGWG mediated the association between lipid levels and SGA [21] (Fig. 1). To determine whether lipids significantly influenced SGA (path c) and iGWG (path a), a log-Poisson regression model was applied. The association between iGWG and SGA (path b) was then examined. Finally, the effect of lipids on SGA while accounting for iGWG as a categorical variable (yes, no) was analysed (path c′). The mediation effect was evaluated using the regression coefficients of pathways a and b (a × b). Analyses were performed using the *mediation* package in R software version 4.3.2 (R Foundation for Statistical Computing, Austria). A *P*-value of < 0.05 was considered statistically significant.

## Results

### Baseline characteristics of the study population

Table 1 summarises the demographic characteristics of the 2,721 participants. The mean pre-pregnancy BMI was 20.8 ± 2.7 kg/m^2^. Overall, 116 (4.26%), 921 (33.85%), and 1,684 (61.89%) participants had inadequate, adequate, and excessive GWG, respectively. Male and female newborns accounted for 54.1% and 45.9% of births, respectively. A total of 262 (9.6%) newborns were classified as SGA. Participants with iGWG were less likely to be primiparous and had a higher prevalence of gestational diabetes mellitus and SGA births.

**Table 1:** Demographic characteristics of the study population stratified by total GWG according to Chinese guidelines^a^

Maternal characteristics	Overall *n* = 2721	Inadequate *n* = 116	Adequate *n* = 921	Excessive *n* = 1684	*P*-value
Age (years)	28.5 ± 3.5	29.6 ± 4.1	28.7 ± 3.4	28.4 ± 3.4	0.001
Height (cm)	160.3 ± 5.0	160.0 ± 4.8	159.9 ± 4.9	160.5 ± 5.0	0.019
Pre-pregnancy BMI (kg/m^2^)	20.8 ± 2.7	20.9 ± 3.8	20.5 ± 2.6	21.0 ± 2.7	< 0.001
Underweight	518 (19.0)	46 (39.7)	240 (26.1)	232 (13.8)	
Normal weight	1874 (68.9)	47 (40.5)	610 (66.2)	1217 (72.3)	
Overweight and Obese	329 (12.1)	32 (19.8)	71 (7.7)	235 (14.0)	
Ethnicity (Han Chinese)	2656 (97.6)	115 (99.1)	897 (97.4)	1644 (97.6)	0.543
Educational level					0.164
< 12 years	356 (13.1)	16 (13.8)	109 (11.8)	231 (13.7)	
12–15 years	742 (27.3)	29 (25.0)	232 (25.2)	481 (28.6)	
≥ 16 years	1547 (56.9)	70 (60.3)	548 (59.5)	929 (55.2)	
Unknown	76 (2.8)	1(0.9)	32 (3.5)	43 (2.6)	
Monthly income (CNY)					0.313
< 5000	1005 (36.9)	45 (38.8)	317 (34.4)	643 (38.2)	
5000–9999	1125 (41.3)	47 (40.5)	391 (42.5)	687 (40.8)	
≥ 10,000	543 (20.0)	21 (18.1)	199 (21.6)	323 (19.2)	
Unknown	48 (1.8)	3 (2.6)	14 (1.5)	31 (1.8)	
Primiparous	2241 (82.4)	81 (69.8)	736 (79.9)	1424 (84.6)	< 0.001
Regular physical activity in early pregnancy (yes)	650 (23.9)	21 (18.1)	223 (24.2)	406 (24.1)	0.325
Drinking before pregnancy (yes)	329 (12.1)	13 (11.2)	105 (11.4)	211 (12.5)	0.671
Smoking before pregnancy (yes)	85 (3.1)	2 (1.7)	21 (2.3)	62 (3.7)	0.098
Hypertensive disorders of pregnancy (yes)	141 (5.2)	5 (4.3)	22 (2.4)	114 (6.8)	< 0.001
Gestational diabetes mellitus (yes)	300 (11.0)	27 (23.3)	120 (13.0)	153 (9.1)	< 0.001
Total weight gain (kg)	15.9 ± 4.7	7.2 ± 2.7	12.2 ± 2.1	18.6 ± 3.5	< 0.001
Gestational age at blood sampling (weeks)	39.4 ± 1.3	39.1 ± 1.3	39.2 ± 1.3	39.5 ± 1.2	< 0.001
Gestational age at delivery	39.46 ± 1.22	39.17 ± 1.18	39.30 ± 1.27	39.56 ± 1.18	< 0.001
Preterm infant	70 (2.6)	5 (4.3)	38 (4.1)	27 (1.6)	< 0.001
TG (mmol/L)	3.29 (1.71)	3.13 (1.82)	3.23 (1.68)	3.33 (1.72)	0.042
TC (mmol/L)	6.16 (1.55)	6.21 (1.59)	6.24 (1.53)	6.10 (1.54)	0.027
HDL-C (mmol/L)	1.78 (0.54)	1.79 (0.52)	1.78 (0.53)	1.78 (0.55)	0.770
LDL-C (mmol/L)	3.43 (1.36)	3.48 (1.40)	3.54 (1.40)	3.37 (1.33)	0.001
Male newborn	1472 (54.1)	67 (57.8)	517 (56.1)	888 (52.7)	0.180
Birth weight (g)	3344.7 ± 417.0	3104.3 ± 349.8	3237.3 ± 377.0	3420 ± 423.1	< 0.001
SGA	262 (9.6)	26 (22.4)	116 (12.6)	120 (7.1)	< 0.001

CNY, Chinese Yuan, 1 CNY ≈ 0,14 US$

*BMI* Body mass index, *TG* Triglycerides, *TC* Total cholesterol, *HDL-C* High-density lipoprotein cholesterol, *LDL-C* Low-density lipoprotein cholesterol, *SGA* Small-for-gestational-age

^a^Non-normally distributed data are expressed as median (IQR), whereas normally distributed data are presented as mean ± SD. Categorical variables are reported as *n* (%)

*P*-values were calculated using the chi-square test, Kruskal–Wallis test and one-way ANOVA for categorical, non-normally distributed and normally distributed variables, respectively

### Associations between lipid profile and birth outcomes

Table 2 presents the RR and aRR, along with 95% CIs, for SGA across tertiles of maternal lipid levels during the third trimester. Compared with the middle tertile (T2), the highest tertile (T3) of HDL-C was associated with an increased risk of SGA (aRR, 1.54; 95% CI, 1.15–2.07). The lowest tertile of TG (T1) showed a marginal, but non-significant association with SGA risk (aRR, 1.27; 95% CI, 0.95–1.70) compared with T2. These findings were consistent after excluding preterm births (2.6% of the total).

**Table 2:** Associations between lipid levels during the third trimester and SGA risk

Lipid profile	N	SGA			
		***n* (%)**	**Crude model (95% CI)**	**Adjusted model (95% CI)**^**1**^	***P*-value**
**TG (mmol/L)**					
< 2.79	906	108 (11.92)	1.30 (0.97, 1.73)	1.27 (0.95, 1.70)	0.556
2.79–3.90	902	83 (9.20)	Ref. (1.00)	Ref. (1.00)	-
≥ 3.91	913	71 (7.78)	0.85 (0.62, 1.16)	0.91 (0.66, 1.26)	0.109
**TC (mmol/L**					
< 5.67	907	85 (9.37)	0.92 (0.68, 1.23)	1.05 (0.78, 1.42)	0.758
5.67–6.65	901	92 (10.21)	Ref. (1.00)	Ref. (1.00)	-
≥ 6.66	913	85 (9.31)	0.91 (0.68, 1.22)	0.88 (0.65, 1.19)	0.391
**HDL-C (mmol/L)**					
< 1.62	912	69 (7.57)	0.90 (0.65, 1.24)	0.95 (0.68, 1.33)	0.772
1.62–1.96	902	76 (8.43)	Ref. (1.00)	Ref. (1.00)	-
≥ 1.97	907	117 (12.90)	1.53 (1.15, 2.04)	1.54 (1.15, 2.07)	0.004
**LDL-C (mmol/L)**					
< 3.05	909	89 (9.79)	1.02 (0.76, 1.38)	1.12 (0.82, 1.51)	0.478
3.05–3.88	898	86 (9.58)	Ref. (1.00)	Ref. (1.00)	-
≥ 3.89	914	87 (9.52)	0.99 (0.74, 1.34)	0.90 (0.66, 1.22)	0.478

*CI* Confidence interval, *SGA* Small-for-gestational-age, *TG* Triglycerides, *TC* Total cholesterol, *HDL-C* High-density lipoprotein cholesterol, *LDL-C* Low-density lipoprotein cholesterol

Ref., reference

^1^Adjusted for maternal education, ethnicity, age, monthly average income, regular physical activity in early pregnancy, pre-pregnancy BMI, parity, gestational age at blood sampling, total weight gain, and neonatal sex

‘*n* (%)’ values represent the number and percentage of SGA births within each category

### Low TG and high HDL-C combination in relation to SGA risk

Given the individual associations of TG and HDL-C with the risk of SGA, potential interactions between TG and HDL-C were examined. A significant interaction was identified between low TG and high HDL-C levels (*P* for interaction = 0.019; Supplementary Table S1). To further examine this combined effect, women were categorised into four groups based on their TG and HDL-C tertiles: (1) middle tertile (T2) for both TG and HDL-C (reference group); (2) low TG (T1) and high HDL-C (T3); (3) high TG (T3) and low HDL-C (T1); and (4) all other combinations. Mothers with low TG and high HDL-C levels had a significantly higher risk of delivering an SGA infant compared with the reference group (aRR, 1.77; 95% CI, 1.10–2.87; Table 3).

**Table 3:** Associations between combined TG and HDL-C levels during the third trimester and SGA risk

Combined TG and HDL-C	N	SGA			
		***n* (%)**	**Crude model (95% CI)**	**Adjusted model (95% CI)**^**1**^	***P*-value**
Middle TG (2.79–3.90) and middle HDL-C (1.62–1.96)	302	24 (7.95)	Ref. (1.00)	Ref. (1.00)	-
Low TG (< 2.79) and high HDL-C (≥ 1.97)	428	63 (14.72)	1.85 (1.16, 2.96)	1.77 (1.10, 2.87)	0.020
High TG (≥ 3.91) and low HDL-C (< 1.62)	430	29 (6.74)	0.85 (0.49, 1.46)	0.94 (0.54, 1.64)	0.839
All other combinations for TG and HDL-C	1561	146 (9.35)	1.18 (0.76, 1.81)	1.19 (0.76, 1.85)	0.447

*CI* Confidence interval, *SGA* Small-for-gestational-age, *TG* Triglycerides, *HDL-C* High-density lipoprotein cholesterol

Ref., reference

^1^Adjusted for maternal education, ethnicity, age, monthly average income, regular physical activity in early pregnancy, pre-pregnancy BMI, parity, gestational age at blood sampling, total weight gain, and neonatal sex

‘*n* (%)’ values represent the number and percentage of SGA births within each category

This association was further confirmed in sensitivity analyses restricted to women with normal pre-pregnancy BMI, which demonstrated an even stronger association (aRR, 2.28; 95% CI, 1.16–4.47; Supplementary Table S2).

### Potential influence of iGWG on the relationship between lipid profiles and SGA risk

GWG has been reported to influence both lipid levels and the risk of SGA. As shown in Table 4, the associations of high HDL-C levels and combined low TG–high HDL-C with SGA remained significant after adjustment for iGWG (path c'). No significant indirect effects through iGWG were observed (a × b). Although iGWG was significantly associated with SGA (path b), it was not associated with maternal lipid levels (path a). The robustness of these findings was supported by sensitivity analyses, excluding women with gestational diabetes mellitus and hypertensive pregnancy disorders (Supplementary Table S3). These results indicate that maternal lipid levels markedly influence foetal growth and SGA risk independent of iGWG.

**Table 4:** Mediation of iGWG in the association between maternal lipid levels and SGA risk

	**Path c**		**Path c'**		**Path a**		**Path b**		**Mediating effect**		
**Lipids**		**Coefficient (95% CI)**		**Coefficient (95% CI)**		**Coefficient (95% CI)**		**Coefficient (95% CI)**		**a × b**	***P*-value**
TG < 2.79		0.209 (− 0.184, 0.610)		0.212 (− 0.180, 0.613)		0.008 (− 0.137, 0.153)		0.690 (0.240, 1.103)*		0.0055	0.914
TG ≥ 3.91		− 0.110 (− 0.566, 0.338)		− 0.126 (− 0.581, 0.323)		0.010 (− 0.138, 0.158)		0.690 (0.240, 1.103)*		0.0068	0.895
HDL-C < 1.62		0.219 (− 0.262, 0.707)		0.249 (− 0.233, 0.736)		− 0.009 (− 0.154, 0.136)		0.666 (0.215, 1.079)*		−0.0058	0.905
HDL-C ≥ 1.97		0.794 (0.379, 1.233)*		0.797 (0.381, 1.236)*		0.003 (− 0.143, 0.148)		0.666 (0.215, 1.079)*		0.0017	0.972
TG (< 2.79) and HDL-C (≥ 1.97)		1.216 (0.468, 2.115)*		1.195 (0.446, 2.094)*		0.036 (− 0.196, 0.271)		0.648 (0.197, 1.061)*		0.0233	0.766

The table presents the coefficients and 95% CIs for each stage of the mediation analysis, estimated using the *mediation* package with log-Poisson models adjusted for maternal ethnicity, education, age, monthly average income, parity, regular physical activity in early pregnancy, pre-pregnancy BMI, gestational age at blood sampling and neonatal sex. Path c represents the association between lipid levels and SGA, whereas path c' represents this association after adjusting for iGWG (yes, no). Path a represents the association between lipid levels and iGWG, and path b represents the association between iGWG and SGA. The significance of a × b was assessed using the Sobel test. * *P* < 0.05

*CI* Confidence interval, *TG* Triglycerides, *HDL-C* High-density lipoprotein cholesterol

## Discussion

This study examined maternal lipid profiles during the third trimester of pregnancy and their association with the risk of delivering SGA newborns. High HDL-C levels were positively associated with an increased SGA risk. Furthermore, an elevated risk of SGA was particularly observed among mothers with low TG and high HDL-C levels. The findings of this study revealed that the combination of low TG and high HDL-C levels during the third trimester was a significant risk factor for SGA infants, suggesting a potential interaction between these lipid components in influencing foetal growth. These associations were independent of iGWG.

Maternal lipid levels may influence the placental vascular endothelium, thereby disrupting placental function and potentially leading to underperfusion and abnormal birth weight. Nevertheless, the precise mechanisms through which maternal lipid profiles affect neonatal outcomes remain unclear. During pregnancy, trophoblasts take up glycerol and free fatty acids released from HDL-C and LDL-C via placental enzyme-catalysed hydrolysis. These absorbed components are subsequently re-esterified to support foetal development [22]. Under normal physiological conditions, HDL-C possesses anti-inflammatory and antioxidant properties that help protect against cardiovascular diseases [23] and play a crucial role in removing cholesterol and other lipids from peripheral tissues [24]. Although this study demonstrated the association between high HDL-C levels and an increased SGA risk, this finding may be explained by alterations in HDL-C function under pathological conditions. Such changes can impair vascular endothelial cell function [25], potentially affecting placental function and foetal growth. Elevated HDL-C concentrations have been associated with unfavourable pregnancy outcomes, including spontaneous preterm delivery [26]. Elevated HDL-C concentrations during pregnancy may reflect placental dysfunction resulting from altered lipid metabolism or peroxidation. This dysfunction may contribute to SGA by reducing the transfer of essential nutrients or by interfering with placental blood flow, thereby disrupting the normal transfer of HDL-C to the foetus. Consistently, SGA infants have been reported to exhibit lower cord blood HDL-C concentrations compared with infants born appropriate for gestational age [27]. Accordingly, elevated maternal HDL-C levels may result from placental dysfunction, which decreases the amount of maternal HDL-C transported to the foetus [28].

Consistent with these findings, a previous study reported that high HDL-C levels during the third trimester were markedly associated with an increased SGA risk among women with appropriate GWG [29]. Additionally, elevated HDL-C levels at 36 weeks of gestation have been associated with an increased risk of developing SGA. Furthermore, reduced newborn size has been linked to alterations in maternal HDL-C levels over the course of pregnancy [30]. Mothers who gave birth to SGA infants exhibited higher HDL-C levels compared with those who delivered normal birth weight infants [28]. However, evidence regarding the relationship between HDL-C levels and birth weight remains inconsistent, with some studies reporting inverse associations, particularly among women classified as overweight or obese [31, 32].

Based on the findings of the present study, TG levels approached statistical significance in relation to SGA, suggesting a potential role of TG in influencing birth weight. Low maternal TG levels during pregnancy were found to be more likely to result in SGA infants, whereas elevated maternal TG levels were associated with a higher risk of large-for-gestational-age (LGA) infants [33]. Furthermore, the prevalence of SGA has demonstrated negative correlation with HDL-C concentrations in underweight women before pregnancy and with TG levels across all BMI groups [5]. In addition, a meta-analysis reported that higher maternal TG levels during pregnancy were associated with a lower risk of SGA [34].

Maternal TG levels are well recognised for their substantial role in foetal development, despite TG being unable to cross the placenta directly. TG is broken down by endothelial-placental lipase, generating free fatty acids that are absorbed by placental trophoblasts. This process, in turn, influences placental development and angiogenesis [35, 36]. However, dysregulation of placental lipid transport may result in an insufficient nutrient and energy supply to the foetus, thereby contributing to SGA outcomes. Fatty acids may act as growth factors and compete with hormones for albumin binding, thereby increasing free hormone levels in the circulation and potentially affecting intrauterine foetal growth [37]. Importantly, free fatty acids can contribute to insulin resistance, which may have detrimental effects on birth outcomes [38]. As insulin resistance increases the activation of amino acid transport systems, it promotes protein synthesis and decreases lipolysis, potentially impairing glucose tolerance [38]. Maternal nutrition plays a pivotal role in foetal development, as it directly affects the supply of essential nutrients and energy. Maternal diet has been reported to influence the regulation of lipid levels [39, 40]. Furthermore, an animal study has suggested that maternal malnutrition during pregnancy can restrict foetal growth [41]. Infants born with SGA may experience inadequate foetal growth owing to inadequate maternal weight gain, which may also lead to inadequate adipose tissue energy storage and insufficient TC and TG delivery to the foetus during late pregnancy [42]. Various mechanisms contribute to SGA outcomes, including placental dysfunction, obstetric complications, and foetal genetic abnormalities. In addition, smoking during pregnancy has been consistently associated with low birth weight [43, 44]. In contrast, some studies have found no significant effect of TG on neonatal birth weight [32] or HDL-C levels [45]. These discrepancies may be attributable to variations in sample size, population characteristics, gestational age at blood sampling, or other factors.

Based on the assessment of the combined effects of TG and HDL-C levels, the co-occurrence of low TG and high HDL-C levels markedly increased the risk of SGA. Nevertheless, this finding highlights the potential relevance of maternal lipid levels in predicting foetal growth, including among women with normal pre-pregnancy BMI. These results also underscore the importance of considering lipid combinations, rather than evaluating individual lipids in isolation, in prenatal risk assessment. Notably, low TG and high HDL-C levels during the third trimester emerged as significant predictors of SGA infants.

As previously noted, the objective of the mediation analysis was to clarify the mechanisms underlying the association between lipid profiles and SGA. iGWG is an important concern during pregnancy owing to its association with several adverse maternal and foetal outcomes. The analysis indicated that the observed associations between high HDL-C, the low TG–high HDL-C combination, and SGA could not be explained by iGWG. This finding suggests that the relationship between lipid levels and SGA is not related to iGWG. These results highlight the potential value of integrating lipid profile monitoring into prenatal care, which may help inform more tailored strategies for managing pregnancy and enhancing foetal well-being.

Thus, monitoring lipid profiles during pregnancy or obtaining lipid measurements before pregnancy may help improve maternal lipid status at an early stage. Given that women undergoing preconception may be more willing to modify their lifestyles, pre-pregnancy lipid level assessment may offer an opportunity to reduce subsequent risks and enable timely intervention [46]. Monitoring lipid levels during pregnancy also provides an opportunity to adapt to lifestyle changes that may improve pregnancy outcomes and support the well-being of mothers and their foetuses. Although therapeutic options for modifying lipid profiles during pregnancy remain limited, these results highlight the need for further research on early identification and intervention, particularly among high-risk women with abnormal lipid profiles. Such efforts may enhance monitoring and personalised care, thereby improving pregnancy outcomes.

### Strengths and limitations

This study has several important implications. It was conducted using standardised data and biological samples collected from a large prospective observational cohort study. Data on lifestyle factors and maternal characteristics during pregnancy were collected and analysed, allowing adjustment for several important confounding factors. In addition, fasting blood samples were obtained in the morning to minimise the influence of non-fasting conditions on lipid levels.

Despite these strengths, some limitations should be considered. Pre-pregnancy weight was self-reported, which may introduce reporting bias. However, this is a common practice and is generally considered acceptable, as weight was measured at enrolment in early pregnancy, when body weight did not change significantly [47]. Additionally, a considerable proportion of participants were Han Chinese, which may limit the generalisability of the findings to other communities or ethnic populations. Given the observational nature of this study, the results may also have been influenced by unmeasured confounding factors, despite adjustment for several known covariates. In particular, unmeasured factors such as dietary intake and maternal metabolic status may have influenced the observed associations. Although genetic risk factors are an important consideration, they were not included in this analysis. Nonetheless, investigating these factors will be the central focus of the next phase of this research. Given the limited sample size with longitudinal lipid data, this study focused on maternal lipid profiles measured during the third trimester. However, analyses of the available data indicated that both baseline lipids and their subsequent changes during gestation strongly predicted third-trimester lipid concentrations. Although repeated measurements would offer a more comprehensive understanding of lipid dynamics in relation to SGA, this study underscores the significant association between third trimester maternal lipid status and SGA.

This study did not perform sensitivity analyses using alternative birth-weight references or growth charts, such as those from the WHO or INTERGROWTH-21ST. However, similar associations employing various reference charts have been reported in earlier literature. For example, a Japanese study [33] identified SGA and LGA infants using Japanese neonatal charts based on gestational ages at birth. The INTERGROWTH-21ST standards for birth weight were used in rural Gambia to define SGA [42]. According to both studies, SGA risk and infant weight at birth correlated with lipid levels throughout pregnancy, supporting the robustness of the findings of the present study. The classification of SGA in this study was based on Chinese reference standards, which are widely used in studies conducted in China and provide relevant context for the study population. However, birth-weight standards vary across regions owing to differences in socioeconomic status, nutritional habits, healthcare access, and environmental conditions. Consequently, the findings of this study may not fully capture the diversity of birth weight distributions across all regions of China.

This study demonstrated that lipid levels were a notable risk factor for SGA among women with normal pre-pregnancy BMI. However, it is important to note that the analyses of the associations between HDL-C and TG levels in the underweight and overweight/obese pre-pregnancy BMI groups were not feasible owing to small sample sizes. In addition, the mediation analysis employed Baron and Kenny’s approach, which remains a widely accepted and clear framework. Although more recent methods, such as the VanderWeele approach, are available, the Baron and Kenny method remains relevant and has been used in a recent study [48]. Further research is needed to clarify the mechanisms through which lipid profiles influence foetal growth outcomes.

## Conclusions

This study demonstrated that an increased SGA risk was associated with high HDL-C levels and that this risk was further elevated when high HDL-C co-occurred with low TG levels. Importantly, the association between maternal lipid profiles and SGA appeared to be independent of iGWG.

Clinically, in addition to managing GWG, it is also important to monitor the lipid profiles during pregnancy, particularly in cases of unexpectedly low TG and high HDL-C levels, to mitigate the risk of SGA.

## Supplementary Information

Supplementary Material 1.

## Data Availability

The datasets used and/or analysed during the current study are available from the corresponding author on reasonable request.

## Abbreviations

aRR: Adjusted relative risk
BMI: Body mass index
CI: Confidence interval
GWG: Gestational weight gain
HDL-C: High-density lipoprotein cholesterol
iGWG: Inadequate gestational weight gain
LDL-C: Low-density lipoprotein cholesterol
RR: Relative risk
SGA: Small-for-gestational-age
TC: Total cholesterol
TG: Triglycerides

## References

[1] Mankuta D, Elami-Suzin M, Elhayani A, Vinker S. Lipid profile in consecutive pregnancies. Lipids Health Dis. 2010;9:1–4. 20525387 10.1186/1476-511X-9-58 PMC2904773

[2] Saarelainen H, Laitinen T, Raitakari OT, Juonala M, Heiskanen N, Lyyra-Laitinen T, et al. Pregnancy-related hyperlipidemia and endothelial function in healthy women. Circ J. 2006;70:768–72. 16723801 10.1253/circj.70.768

[3] Wang W, Li N, Wang X, Zhang X, Tu M, Lin L, et al. Remnant cholesterol is associated with gestational diabetes mellitus: a cohort study. J Clin Endocrinol Metab. 2023;dgad262. 10.1210/clinem/dgad262 37167108

[4] Yang Y, Wang Y, Lv Y, Ding H. Dissecting the roles of lipids in preeclampsia. Metabolites. 2022;12:590. 35888713 10.3390/metabo12070590 PMC9323219

[5] Zhu SM, Zhang HQ, Li C, Zhang C, Yu JL, Wu YT, et al. Maternal lipid profile during early pregnancy and birth weight: a retrospective study. Front Endocrinol (Lausanne). 2022;13:951871. 36187100 10.3389/fendo.2022.951871 PMC9521310

[6] Shi P, Tang J, Yin X. Association between second-and third-trimester maternal lipid profiles and adverse perinatal outcomes among women with GDM and non-GDM: a retrospective cohort study. BMC Pregnancy Childbirth. 2023;23(1):318. 37147564 10.1186/s12884-023-05630-5 PMC10161404

[7] Ludvigsson JF, Lu D, Hammarström L, Cnattingius S, Fang F. Small for gestational age and risk of childhood mortality: a Swedish population study. PLoS Med. 2018;15:e1002717. 30562348 10.1371/journal.pmed.1002717 PMC6298647

[8] Liu Q, Yang H, Sun X, Li G. Risk factors and complications of small for gestational age. Pak J Med Sci. 2019;35:1199. 31488978 10.12669/pjms.35.5.253 PMC6717459

[9] Yu B, Garcy AM. A longitudinal study of cognitive and educational outcomes of those born small for gestational age. Acta Paediatr. 2018;107:86–94. 28712154 10.1111/apa.13993 PMC5763381

[10] Zamojska J, Niewiadomska-Jarosik K, Kierzkowska B, Gruca M, Wosiak A, Smolewska E. Lipid profile in children born small for gestational age. Nutrients. 2023;15:4781. 38004175 10.3390/nu15224781 PMC10674326

[11] Oldereid NB, Wennerholm U-B, Pinborg A, Loft A, Laivuori H, Petzold M, et al. The effect of paternal factors on perinatal and paediatric outcomes: a systematic review and meta-analysis. Hum Reprod Update. 2018;24:320–89. 29471389 10.1093/humupd/dmy005

[12] Obesity LP-M, Voerman E, Santos S, Inskip H, Amiano P, Barros H, et al. Association of gestational weight gain with adverse maternal and infant outcomes. JAMA. 2019;321:1702. 10.1001/jama.2019.3820 PMC6506886 31063572

[13] Kozuki N, Katz J, Lee ACC, Vogel JP, Silveira MF, Sania A, et al. Short maternal stature increases risk of small-for-gestational-age and preterm births in low-and middle-income countries: individual participant data meta-analysis and population attributable fraction. J Nutr. 2015;145:2542–50. 26423738 10.3945/jn.115.216374 PMC6457093

[14] Wang Y, Chen Z, Zhang F. Association between maternal lipid levels during pregnancy and delivery of small for gestational age: A systematic review and meta-analysis. Front Pediatr. 2022;10:934505. 10.3389/fped.2022.934505 PMC9582334 36275062

[15] Liu X, Wang H, Yang L, Zhao M, Magnussen CG, Xi B. Associations between gestational weight gain and adverse birth outcomes: a population-based retrospective cohort study of 9 million mother-infant pairs. Front Nutr. 2022;9:811217. 35237640 10.3389/fnut.2022.811217 PMC8882729

[16] Zhang K, Jia X, Yu W, Cheng X, Li Y, Wang X, et al. The associations of gestational weight gain and midpregnancy lipid levels with placental size and placental-to-birth weight ratio: findings from a Chinese birth cohort study. BMC Pregnancy Childbirth. 2023;23:725. 37821857 10.1186/s12884-023-05991-x PMC10568921

[17] Zhong C, Chen R, Zhou X, Zhang Y, Liu C, Huang L, et al. Cohort Profile: The Tongji Maternal and Child Health Cohort (TMCHC). Int J Epidemiol. 2023;52:e152–61. 36343093 10.1093/ije/dyac209

[18] Dai L, Deng C, Li Y, Zhu J, Mu Y, Deng Y, et al. Birth weight reference percentiles for Chinese. PLoS ONE. 2014;9:e104779. 25127131 10.1371/journal.pone.0104779 PMC4134219

[19] Zhou B. Predictive values of body mass index and waist circumference to risk factors of related diseases in Chinese adult population. Zhonghua Liu Xing Bing Xue Za Zhi. 2002;23:5–10. 12015100

[20] Society CN. Weight Monitoring Evaluation During Pregnancy Period of Chinese Women. 2021; https://www.cnsoc.org/otherNotice/392100200.html. Accessed Sept 2024.

[21] Baron RM, Kenny DA. The moderator–mediator variable distinction in social psychological research: conceptual, strategic, and statistical considerations. J Pers Soc Psychol. 1986;51:1173. 3806354 10.1037//0022-3514.51.6.1173

[22] von Versen-Hoeynck FM, Powers RW. Maternal-fetal metabolism in normal pregnancy and preeclampsia. Front Biosci. 2007;12:2457–70. 17127255 10.2741/2247

[23] Shuhei N, Söderlund S, Jauhiainen M, Taskinen M-R. Effect of HDL composition and particle size on the resistance of HDL to the oxidation. Lipids Health Dis. 2010;9:1–10. 20863394 10.1186/1476-511X-9-104 PMC2954910

[24] Brown WV. High-density lipoprotein and transport of cholesterol and triglyceride in blood. J Clin Lipidol. 2007;1:7–19. 21291664 10.1016/j.jacl.2007.02.001

[25] Liu X, Wang TT, Li Y, Shi MM, Li HM, Yuan HX, et al. High density lipoprotein from coronary artery disease patients caused abnormal expression of long non-coding RNAs in vascular endothelial cells. Biochem Biophys Res Commun. 2017;487:552–9. 28427943 10.1016/j.bbrc.2017.04.082

[26] Chen X, Scholl TO, Stein TP, Steer RA, Williams KP. Maternal circulating lipid profile during early pregnancy: racial/ethnic differences and association with spontaneous preterm delivery. Nutrients. 2017;9:19. 28045435 10.3390/nu9010019 PMC5295063

[27] Kwiterovich PO, Cockrill SL, Virgil DG, Garrett ES, Otvos J, Knight-Gibson C, et al. A large high-density lipoprotein enriched in apolipoprotein CI: a novel biochemical marker in infants of lower birth weight and younger gestational age. JAMA. 2005;293:1891–9. 15840864 10.1001/jama.293.15.1891

[28] Kramer MS, Kahn SR, Dahhou M, Otvos J, Genest J, Platt RW, et al. Maternal lipids and small for gestational age birth at term. J Pediatr. 2013;163:983–8. 23810722 10.1016/j.jpeds.2013.05.014

[29] Chen Q, Chen H, Xi F, Sagnelli M, Zhao B, Chen Y, et al. Association between maternal blood lipids levels during pregnancy and risk of small-for-gestational-age infants. Sci Rep. 2020;10:1–12. 33199750 10.1038/s41598-020-76845-1 PMC7669834

[30] Wang H, Dang Q, Zhu H, Liang N, Le Z, Huang D, et al. Associations between maternal serum HDL-c concentrations during pregnancy and neonatal birth weight: a population-based cohort study. Lipids Health Dis. 2020;19:1–9. 32410711 10.1186/s12944-020-01264-0 PMC7227214

[31] Misra VK, Trudeau S, Perni U. Maternal serum lipids during pregnancy and infant birth weight: the influence of prepregnancy BMI. Obesity. 2011;19:1476–81. 21394096 10.1038/oby.2011.43

[32] Sommer C, Sletner L, Mørkrid K, Jenum AK, Birkeland KI. Effects of early pregnancy BMI, mid-gestational weight gain, glucose and lipid levels in pregnancy on offspring’s birth weight and subcutaneous fat: a population-based cohort study. BMC Pregnancy Childbirth. 2015;15:1–9. 25879215 10.1186/s12884-015-0512-5 PMC4424559

[33] Go H, Hashimoto K, Maeda H, Ogasawara K, Kyozuka H, Murata T, et al. Maternal triglyceride levels and neonatal outcomes: the Japan environment and children’s study. J Clin Lipidol. 2023;17:356–66. 37210241 10.1016/j.jacl.2023.04.005

[34] Wang J, Moore D, Subramanian A, Cheng KK, Toulis KA, Qiu X, et al. Gestational dyslipidaemia and adverse birthweight outcomes: a systematic review and meta-analysis. Obes Rev. 2018;19:1256–68. 29786159 10.1111/obr.12693

[35] Brett KE, Ferraro ZM, Yockell-Lelievre J, Gruslin A, Adamo KB. Maternal–fetal nutrient transport in pregnancy pathologies: The role of the placenta. Int J Mol Sci. 2014;15:16153–85. 25222554 10.3390/ijms150916153 PMC4200776

[36] Szabo AJ. Transferred maternal fatty acids stimulate fetal adipogenesis and lead to neonatal and adult obesity. Med Hypotheses. 2019;122:82–8. 30593430 10.1016/j.mehy.2018.10.022

[37] Fasshauer M, Seeger J, Waldeyer T, Schrey S, Ebert T, Kratzsch J, et al. Serum levels of the adipokine adipocyte fatty acid–binding protein are increased in preeclampsia. Am J Hypertens. 2008;21:582–6. 18437151 10.1038/ajh.2008.23

[38] Chavan-Gautam P, Rani A, Freeman DJ. Distribution of fatty acids and lipids during pregnancy. Adv Clin Chem. 2018;84:209–39. 29478515 10.1016/bs.acc.2017.12.006

[39] Herrera E. Implications of dietary fatty acids during pregnancy on placental, fetal and postnatal development—a review. Placenta. 2002;23:S9-19. 11978055 10.1053/plac.2002.0771

[40] Van Der Pligt PF, Kuswara K, McNaughton SA, Abbott G, Islam SMS, Huynh K, et al. Maternal diet quality and associations with plasma lipid profiles and pregnancy-related cardiometabolic health. Eur J Nutr. 2023;62:3369–81. 37646831 10.1007/s00394-023-03244-3 PMC10611854

[41] Zhu W, Tang S, Shen Z, Wang Y, Liang L. Growth hormone reverses dyslipidemia in adult offspring after maternal undernutrition. Sci Rep. 2017;7:6038. 28729704 10.1038/s41598-017-05045-1 PMC5519748

[42] Okala SG, Sise EA, Sosseh F, Prentice AM, Woollett LA, Moore SE. Maternal plasma lipid levels across pregnancy and the risks of small-for-gestational age and low birth weight: a cohort study from rural Gambia. BMC Pregnancy Childbirth. 2020;20:1–16. 10.1186/s12884-020-2834-1 PMC7068879 32164563

[43] Finken MJJ, van der Steen M, Smeets CCJ, Walenkamp MJE, de Bruin C, Hokken-Koelega ACS, et al. Children born small for gestational age: differential diagnosis, molecular genetic evaluation, and implications. Endocr Rev. 2018;39:851–94. 29982551 10.1210/er.2018-00083

[44] Garrabou G, Hernàndez A, Catalan Garcia M, Morén C, Tobías E, Córdoba S, et al. Molecular basis of reduced birth weight in smoking pregnant women: mitochondrial dysfunction and apoptosis. Addiction Biol. 2016;21:159–70. 10.1111/adb.12183 25186090

[45] Emet T, Üstüner I, Güven SG, Balık G, Ural ÜM, Tekin YB, et al. Plasma lipids and lipoproteins during pregnancy and related pregnancy outcomes. Arch Gynecol Obstet. 2013;288:49–55. 23400357 10.1007/s00404-013-2750-y

[46] Stephenson J, Heslehurst N, Hall J, Schoenaker DAJM, Hutchinson J, Cade JE, et al. Before the beginning: nutrition and lifestyle in the preconception period and its importance for future health. Lancet. 2018;391:1830–41. 29673873 10.1016/S0140-6736(18)30311-8 PMC6075697

[47] Lin L, Chen X, Zhong C, Huang L, Li Q, Zhang X, et al. Timing of gestational weight gain in association with birth weight outcomes: a prospective cohort study. Br J Nutr. 2022. 10.1017/s0007114522001921. 35848157 10.1017/S0007114522001921

[48] Hu H, Feng P, Yu Q, Zhu W, Xu H, Wu D, et al. The mediating role of gestational diabetes mellitus in the associations of maternal prepregnancy body mass index with neonatal birth weight. J Diabetes. 2022;14:26–33. 34668330 10.1111/1753-0407.13233 PMC9060130

