# Supplementary material for: Relationship between maternal lipid profile during the third trimester and the risk of small-for-gestational-age birth: exploring inadequate gestational weight gain as a mediator
Source: Lipids Health Dis. 2026 Jan 12;25:44. doi: 10.1186/s12944-026-02865-x (PMC12888231; doi:10.1186/s12944-026-02865-x)
Supplement: Supplementary file 1 — Supplementary Material 1. [file 12944_2026_2865_MOESM1_ESM.docx]

**Supplementary Materials**

| **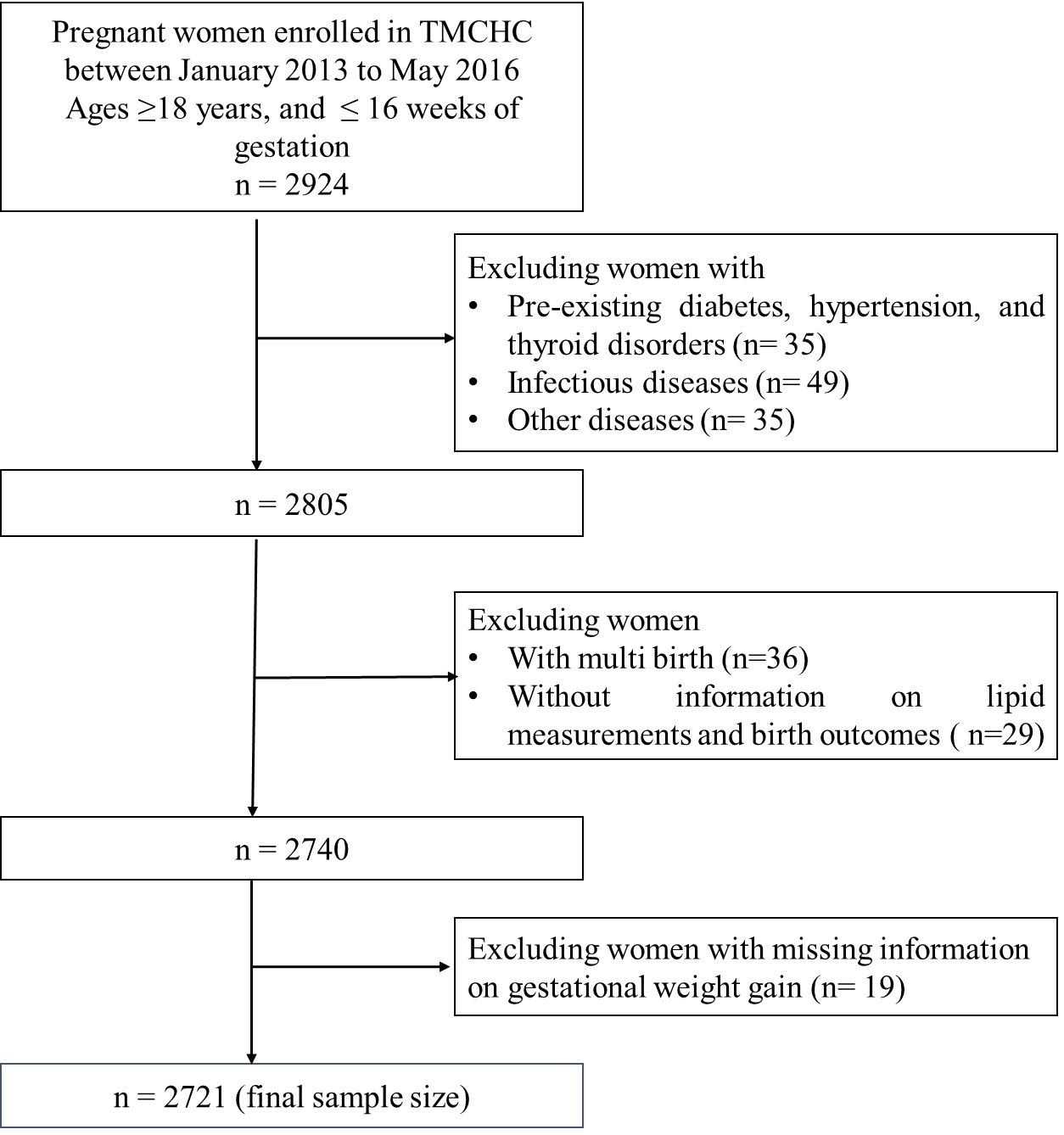** |
| --- |
| **Fig. S1** Flowchart of the study population |

| **Table S1** Interaction between TG and HDL-C levels during the third trimester and the SGA risk | | | | | |
| --- | --- | --- | --- | --- | --- |
| **Interaction of TG and HDL-C** | **N** | **SGA** | | | |
|  |  | ***n* (%)** | **Crude model (95% CI)** | **Adjusted model (95% CI)^1^** | ***P*-interaction** |
| TG (2.79–3.90) * HDL-C (1.62–1.96) | 302 | 24 (7.95) | Ref. (1.00) | Ref. (1.00) | - |
| TG (2.79–3.90) * HDL-C (< 1.62) | 296 | 23 (7.77) | 0.98 (0.55, 1.73) | 0.97 (0.54, 1.75) | 0.915 |
| TG (2.79–3.90) * HDL-C (≥ 1.97) | 304 | 36 (11.84) | 1.49 (0.89, 2.50) | 1.48 (0.87, 2.51) | 0.146 |
| TG (< 2.79) * HDL-C (1.62–1.96) | 292 | 28 (9.59) | 1.21 (0.70, 2.08) | 1.20 (0.69, 2.10) | 0.516 |
| TG (< 2.79) * HDL-C (< 1.62) | 186 | 17 (9.14) | 1.15 (0.62, 2.14) | 1.17 (0.62, 2.19) | 0.631 |
| TG (< 2.79) * HDL-C (≥ 1.97) | 428 | 63 (14.72) | 1.85 (1.16, 2.96) | 1.78 (1.10, 2.88) | 0.019 |
| TG (≥ 3.91) * HDL-C (1.62–1.96) | 308 | 24 (7.79) | 0.98 (0.56, 1.73) | 0.96 (0.54, 1.72) | 0.898 |
| TG (≥ 3.91) * HDL-C (< 1.62) | 430 | 29 (6.74) | 0.85 (0.49, 1.46) | 0.94 (0.54, 1.64) | 0.834 |
| TG (≥ 3.91) * HDL-C (≥ 1.97) | 175 | 18 (10.29) | 1.29 (0.70, 2.39) | 1.46 (0.78, 2.71) | 0.234 |
| SGA: small-for-gestational-age, TG: triglycerides, HDL-C: high-density lipoprotein cholesterol  Ref., reference  ^1^Adjusted for maternal age, ethnicity, education, monthly average income, parity, regular physical activity in early pregnancy, gestational age at blood sampling, total weight gain, and neonatal sex  **Note**: The ‘n (%)’ values represent the number and percentage of SGA births within each category | | | | | |

| **Table S2** Association between combined TG and HDL-C levels during the third trimester and the SGA risk in normal pre-pregnancy BMI | | | | | |
| --- | --- | --- | --- | --- | --- |
| **Combined of TG and HDL-C** | **N** | **SGA** | | | |
|  |  | ***n* (%)** | **Crude model (95% CI)** | **Adjusted model (95% CI)^1^** | ***P*- value** |
| Middle TG (2.79–3.90) and middle HDL-C (1.62–1.96) | 192 | 12 (6.25) | Ref. (1.00) | Ref. (1.00) | - |
| Low TG (<2.79) and high HDL-C (≥1.97) | 299 | 40 (13.38) | 2.14 (1.12, 4.08) | 2.28 (1.16, 4.47) | 0.017 |
| High TG (≥3.91) and low HDL-C (<1.62) | 312 | 20 (6.41) | 1.03 (0.50, 2.10) | 1.12 (0.53, 2.36) | 0.763 |
| All other combinations for TG and HDL-C | 1071 | 80 (7.47) | 1.19 (0.65, 2.19) | 1.28 (0.68, 2.40) | 0.452 |
| SGA: small-for-gestational-age, TG: triglycerides, HDL-C: high-density lipoprotein cholesterol  Ref., reference  ^1^Adjusted for maternal age, ethnicity, education, monthly average income, parity, regular physical activity in early pregnancy, gestational age at blood sampling, total weight gain, and neonatal sex  Note: The ‘*n* (%)’ values represent the number and percentage of SGA births within each category | | | | | |

| **Table S3** Mediation of iGWG on the association between maternal lipid levels during the third trimester and the SGA risk (in sensitivity analysis, women with hypertensive disorders of pregnancy and gestational diabetes mellitus were excluded). | | | | | | |
| --- | --- | --- | --- | --- | --- | --- |
|  | **Path c** | **Path c'** | **Path a** | **Path b** | **Mediating effect** | |
| **Lipids** | **Coefficient**  **(95 % CI)** | **Coefficient**  **(95 % CI)** | **Coefficient**  **(95 % CI)** | **Coefficient**  **(95 % CI)** | **a ×**  **b** | ***P*-**  **value** |
| TG < 2.79 | 0.288 (-0.135, 0.725) | 0.285 (-0.138, 0.722) | 0.002 (-0.157, 0.162) | **0.767 (0.275, 1.214)*** | 0.0017 | 0.978 |
| TG ≥ 3.91 | 0.001 (-0.487, 0.487) | -0.023 (-0.512, 0.463) | 0.005 (-0.159, 0.170) | **0.767 (0.275, 1.214)*** | 0.0041 | 0.949 |
| HDL-C < 1.62 | 0.186 (-0.336, 0.713) | 0.221 (-0.302, 0.749) | -0.019 (-0.182, 0.142) | **0.706 (0.212, 1.155)*** | -0.0140 | 0.810 |
| HDL-C ≥ 1.97 | **0.772 (0.330, 1.241)*** | **0.755 (0.313, 1.225)*** | 0.020 (-0.138, 0.178) | **0.706 (0.212, 1.155)*** | 0.0141 | 0.805 |
| TG (< 2.79) and HDL-C (≥ 1.97) | **1.150 (0.352, 2.126)*** | **1.122 (0.322, 2.099)*** | 0.035 (-0.222, 0.297) | **0.723 (0.230, 1.171)*** | 0.0253 | 0.794 |
| TG: triglycerides, HDL-C: high-density lipoprotein cholesterol  **Note**: The table presents the coefficients and 95% CI for every stage of mediation analysis using the *mediation* package based on log-Poisson model after adjusting for maternal age, ethnicity, education, monthly average income, parity, pre-pregnancy BMI, regular physical activity in early pregnancy, gestational age at blood sampling and neonatal sex. **Path** **c** reports coefficients and 95% CI of association between lipids and SGA, **path** **c'** reports coefficients and 95% CI after adjust for iGWG (yes, no) in addition to the covariate, **path** **a** reports coefficients and 95% CI of the association between lipids and iGWG, and **path** **b** reports coefficients and 95% CI of iGWG to SGA. The significance of a × b was calculated using the Sobel test. * *P* < 0.05 | | | | | | |
